# Surface functionalization of polyurethane scaffolds mimicking the myocardial microenvironment to support cardiac primitive cells

**DOI:** 10.1371/journal.pone.0199896

**Published:** 2018-07-06

**Authors:** Monica Boffito, Franca Di Meglio, Pamela Mozetic, Sara Maria Giannitelli, Irene Carmagnola, Clotilde Castaldo, Daria Nurzynska, Anna Maria Sacco, Rita Miraglia, Stefania Montagnani, Nicoletta Vitale, Mara Brancaccio, Guido Tarone, Francesco Basoli, Alberto Rainer, Marcella Trombetta, Gianluca Ciardelli, Valeria Chiono

**Affiliations:** 1 Department of Mechanical and Aerospace Engineering, Politecnico di Torino, Turin, Italy; 2 Department of Public Health, University of Naples ‘Federico II’, Naples, Italy; 3 Department of Engineering, Tissue Engineering Unit, Università Campus Bio-Medico di Roma, Rome, Italy; 4 Center for Translational Medicine, International Clinical Research Center, St.Anne’s University Hospital, Brno, Czechia; 5 Department of Molecular Biotechnology and Health Sciences, University of Turin, Turin, Italy; 6 Institute for Photonics and Nanotechnology, National Research Council, Rome, Italy; University of Central Florida, UNITED STATES

## Abstract

Scaffolds populated with human cardiac progenitor cells (CPCs) represent a therapeutic opportunity for heart regeneration after myocardial infarction. In this work, square-grid scaffolds are prepared by melt-extrusion additive manufacturing from a polyurethane (PU), further subjected to plasma treatment for acrylic acid surface grafting/polymerization and finally grafted with laminin-1 (PU-LN1) or gelatin (PU-G) by carbodiimide chemistry. LN1 is a cardiac niche extracellular matrix component and plays a key role in heart formation during embryogenesis, while G is a low-cost cell-adhesion protein, here used as a control functionalizing molecule. X-ray photoelectron spectroscopy analysis shows nitrogen percentage increase after functionalization. O1s and C1s core-level spectra and static contact angle measurements show changes associated with successful functionalization. ELISA assay confirms LN1 surface grafting. PU-G and PU-LN1 scaffolds both improve CPC adhesion, but LN1 functionalization is superior in promoting proliferation, protection from apoptosis and expression of differentiation markers for cardiomyocytes, endothelial and smooth muscle cells. PU-LN1 and PU scaffolds are biodegraded into non-cytotoxic residues. Scaffolds subcutaneously implanted in mice evoke weak inflammation and integrate with the host tissue, evidencing a significant blood vessel density around the scaffolds. PU-LN1 scaffolds show their superiority in driving CPC behavior, evidencing their promising role in myocardial regenerative medicine.

## Introduction

Coronary artery disease is the leading mortality cause in the world with more than 4 million Europeans dying every year [1]. Despite the significant advances in pharmaceutical and interventional approaches, existing therapies cannot compensate for cardiomyocyte loss and myocardial function decrease. In the last decades, several groups have identified and isolated resident cardiac primitive cells (CPCs) in the heart tissue [2–5], using a variety of cell surface markers, among which the stem cell factor receptor c-kit (CD117) [6–8]. Since then, the possibility of myocardial regeneration driven by resident or implanted CPCs has appeared as a new intriguing strategy. However, cell therapies using different cell types including CPCs [9] have shown poor cell retention and survival at the implant site and limited improvement of heart contractility attributed to a paracrine effect exerted by the implanted cells on the resident cells [10, 11]. Scaffold-based strategies are also under investigation for myocardial regeneration and could be exploited to develop CPC-populated patches or scaffolds able to recruit endogenous CPCs [12, 13]. According to the general principle of tissue engineering (TE), 3D scaffolds with biomimetic mechanical, structural and chemical properties respect to the natural extracellular matrix (ECM) should induce stem cells to attach, proliferate and differentiate into the desired cell phenotypes [14]. Stem cells exist *in vivo* in a specialized microenvironment, the stem cell niche, controlling their proliferation and differentiation [15, 16]. Hence, a promising approach to regulate CPC behavior is to engineer scaffolds mimicking the cardiac niche ECM. Mauretti and colleagues have recently published a review focused on the definition and properties of resident CPCs, highlighting the key components of CPC niches and the interplay of CPCs with niche constituents [17]. In 2012, Kim et al. first exploited nanofabrication technologies to design surface nanopatterned dense films mimicking cardiac matrix topographical properties, thus creating a stem cell niche *in vitro* and increasing the myogenic differentiation of progenitor cells cultivated on the developed substrates [18]. A further step in the thorough understanding of the key role exerted by nanotopography on cardiac cell behavior has been done by Mengstead and colleagues that demonstrated the capability of cardiomyocytes to align their contractile direction to substrate nanotopography and reorganize it in response to matrix topography changes [19]. The combination of anisotropic topography and electrical conductivity has recently shown to increase the expression of cell-cell coupling and calcium handling proteins, action potential duration, peak calcium release [20]. In addition to surface topography, CPC culture in a three-dimensional environment has been demonstrated to enhance the expression of cardiac transcription factors compared to traditional 2D culture conditions [21–25]. From a biochemical point of view, Castaldo et al. [26] have recently found that human cardiac fibroblasts (CFs) isolated from human heart tissue can deposit *in vitro* a “biomatrix”, with chemical properties similar to the cardiac niche ECM. Studies on the interaction of CPCs with the “biomatrix” constituent proteins have shown that laminin-1 (LN1) protects CPCs from apoptosis and stimulates their proliferation [27]. LN1 has important role for heart development during embryogenesis [28] and has been found to enhance Caco-2 cell differentiation [29]. Up to now, LN1 has been widely used in TE, especially for nerve regeneration [30, 31], while no literature exists on its use for scaffold functionalization in the field of myocardial TE to promote CPC differentiation, as proposed in this work.

In cardiac TE, the use of CPCs isolated from adult tissues or derived from pluripotent stem cells is still limited [23–25, 32, 33]. Particularly, for CPCs derived from embryonic or induced pluripotent stem cells, safety concerns also arise due to the possible uncontrolled differentiation of any residual pluripotent stem cells. Hence, an accurate control of the differentiation protocol of pluripotent stem cells is needed [34].

Among the previous studies on cardiac TE strategies exploiting CPC potentialities, Forte et al. have investigated the effects of microfabricated scaffold topography on CPC differentiation in the presence of a medium enriched with cardiomyocyte-differentiation factors [23]. Gaetani et al. have fabricated bioprinted alginate constructs encapsulating CPCs, demonstrating partial differentiation of CPCs to cardiomyocytes *in vitro* [24]. Cristallini et al. have reported that bioartificial scaffolds based on poly(3-hydroxybutyrate-co-3-hydroxyvalerate)/gelatin with a biomimetic architecture induce the partial differentiation of cardiac resident cells into the cardiac lineage *in vitro* [25]. Recently, we have prepared scaffolds based on an elastomeric polyurethane (PU) by melt-extrusion additive manufacturing (AM), which showed the ability to support CPC adhesion and viability, while they were unable to stimulate CPC proliferation [35]. The aim of the present work was to investigate the effects of PU scaffolds surface functionalized with LN1 (PU-LN1 scaffolds) on CPC response, in the future perspective of a CPC therapy exploiting scaffolds. LN1 was chosen to provide PU scaffolds with biomimetic cues of the cardiac ECM, while gelatin (G) -another ECM derivative widely used in TE, but not providing cardiac-specific cues- was used as a control functionalizing molecule for PU scaffolds (PU-G scaffolds). Successful surface functionalization was assessed by X-ray Photoelectron Spectroscopy (XPS), colorimetric assays (toluidine blue assay, Enzyme-linked immunosorbent (ELISA) assay) and surface wettability measurements. Accelerated *in vitro* degradation tests were carried out with lipase to prove scaffold degradability and assess the effects of surface functionalization on degradation kinetics; the cytocompatibility of scaffold degradation products was also demonstrated according to ISO rules (ISO 10993–5). CPC adhesion, proliferation and apoptosis on PU, PU-LN1 and PU-G scaffolds was assessed by morphological evaluation and by identifying and quantifying actively cycling Ki67-positive cells. CPC differentiation was evaluated through the expression of typical markers of primitive cardiomyocyte, endothelial cell and smooth muscle cell lineages by Real-Time Quantitative Reverse Transcription-Polymerase Chain Reaction (RT-PCR). Finally, PU and PU-LN1 scaffolds were subcutaneously implanted in mice to assess their biocompatibility and evaluate tissue response to the implantation of newly designed porous matrices. Furthermore, the effects of surface functionalization on body reaction (e.g. inflammation, integration, vascularisation) were also studied.

The innovative contribution of this work was the use of biomimetic scaffold functionalization with LN1, rather than a differentiation medium [23–25, 32] or biomimetic anisotropic architecture [25] to enhance CPC survival and differentiation potential.

## Experimental

### Polyurethane synthesis

A thermoplastic polyurethane (PU; M_n_: 80000 Da; M_w_/M_n_: 1.5) was synthesized from PCL diol (Across Organics, Germany, M_n_ = 2000 Da), 1,4-butane diisocyanate (BDI) (AlloraChem, Italy) and l-lysine ethyl ester dihydrochloride (Sigma-Aldrich, Italy) chain extender, according to a method described in previous publications [35, 36].

#### Scaffold and film preparation

Three-dimensional scaffolds were fabricated using a custom-designed AM equipment [37, 38], as previously described by Chiono et al. [35]. The apparatus comprises a heated dispensing head terminated with a nozzle, a X–Y motorized stage for the positioning of the dispensing head, and a Z-axis for controlling its distance from the stage. The extrusion process was performed by dispensing the polymer in a molten form through a 150 μm nozzle at 8 bar, 155°C, with a 2 mm·s^-1^ relative speed between the nozzle and the X-Y stage. Generation of the process tool-path was performed starting from a CAD geometry using a dedicated software interface. Scaffolds with a homogeneous grid structure were fabricated by depositing two layers of fibers laminated in a 0/90° pattern. For each layer, the inter-axial fiber spacing was 500 μm. PU film samples were prepared by melt-compression as previously described by Boffito el al. [39] and used for the optimization of the grafting protocols.

#### Protein functionalization

Round shaped PU films and scaffolds (6 mm in diameter) were functionalized with G (type A from porcine skin, Sigma-Aldrich, Italy) and mouse LN1 (from Murine Engelbreth-Holm-Swarm tumor, Cultrex, USA) according to the following method, based on a two-step plasma treatment (Diener Electronic, Pico Low Pression Plasma System, Germany) [40]. Initially, the samples were treated with Argon plasma (50 W, 0.7 mbar, 20 sccm) for 5 min to create radical species on their surface. Then, they were immediately subjected to acrylic acid (Sigma-Aldrich, Italy) plasma treatment (50 W, 0.05 mbar, 45 sccm) for 15 min with the aim to graft and polymerize acrylic acid on their surface. By this method, the surface was enriched with -COOH groups, which were then exploited for the covalent grafting of G and LN1. To this purpose, samples were placed in an aqueous solution (pH 5.0) containing 5 mg/mL of 1-ethyl-3-(3-dimethylaminopropyl) carbodiimide (EDC; Sigma-Aldrich, Italy) and 1.25 mg/mL of N-hydroxysuccinimide (NHS; Sigma-Aldrich, Italy) at 4°C for 20 h [40]. Scaffolds were then washed 3 times with deionized water (DIW). Finally, LN1 and G were covalently grafted on previously activated sample surfaces by 20 h incubation in G and LN solutions (10 μg/ml) in phosphate buffered saline (PBS; Sigma-Aldrich, Italy) at room temperature. Samples were withdrawn and rinsed three times with DIW.

### Physico-chemical characterizations of polyurethane scaffolds

#### Morphological characterization

Scaffold morphology was analyzed by FEG-SEM (LEO Supra 1535). Samples were mounted on aluminum stubs, coated with a conductive layer of sputtered gold (Emitech K550 sputter coater) and observed at 5 kV accelerating voltage. Average filament diameter and spacing were calculated from SEM images (IMAGEJ; National Institutes of Health, Bethesda, MD, USA) and expressed as a mean value ± standard deviation (n>20).

#### Chemical surface characterization

To assess the successful surface functionalization, XPS was performed on pristine PU, PU after plasma-polymerized acrylic acid coating (plasma-treated PU), PU-G and PU-LN1 scaffolds using a PHI 5000 Versaprobe instrument in a high vacuum chamber at a power of 25.6 W and a voltage of 23.5 eV, with a scanning area of 100x100 μm^2^. Analysis of XPS spectra was performed using XPSPEAK 4.1 software. TBO colorimetric assay was performed to quantify the–COOH groups on the surface of plasma-treated PU compared to pristine PU [41, 42]. Samples were soaked into 500 μM TBO (Sigma-Aldrich, Italy) aqueous solution (pH 10). The formation of ionic complexes between -COOH groups and the cationic dye was allowed to proceed for 12 hours at room temperature. Samples were then rinsed with 0.1 mM NaOH solution (pH 10) to remove unbound TBO molecules. The bonded TBO was desorbed by incubation in 50% acetic acid solution for 25 min. Solution absorbance at 632 nm was recorded in an UV-Vis spectrophotometer (PerkinElmer, Lambda 25). The amount of–COOH groups was calculated by a calibration curve derived from TBO solutions in acetic acid/water (50% v/v) at selected concentrations in the 2.5 ÷ 25 μM range, and based on the assumption that 1 mol TBO interacts with 1 mol–COOH. Experiments were performed in quintuplicate and results are reported as mean value ± standard deviation. Finally, surface functionalization with LN1 was also evaluated through Enzyme-linked immunosorbent (ELISA) assay. To obtain a standard curve, different wells of microtiter plates were coated with an amount of LN1 solution in PBS with increasing concentrations (0.025, 0.05, 0.075, 0.1, 0.25, 0.5, 0.75 and 1 mg/mL) overnight at 4°C. Coated wells of microtiter plates and PU and PU-LN1 scaffolds were washed three times with PBS and then incubated with PBS containing 5% bovine serum albumin (BSA; Sigma-Aldrich, Italy) at room temperature for 1 h. After washing, they were incubated with rabbit anti-laminin 1 antibody (Abcam ab11575, UK) at 1:10,000 dilution in PBS containing 5% BSA for 1 h. After washing, samples were incubated with a horseradish perodixase (HRP)-coupled goat anti-rabbit antibody (A6154; Sigma-Aldrich, Italy) 1:800 diluted in 5% BSA solution in PBS for 1 h. 90 μl of a 3,3’,5,5′-tetramethylbenzidine (TMB; T0440, Sigma-Aldrich, Italy) solution in dimethyl sulfoxide (DMSO, Sigma-Aldrich, Italy) was added to each well, and the reaction was stopped after 2 min by addition of 90 mL of 0.5 N HCl (Sigma-Aldrich, Italy). Absorbance was read at 450 nm in a microtiter plate reader (GloMax-Multidetection System, Promega). The standard curve was generated using a second order polynomial equation using GraphPad Prism (GraphPad Software, La Jolla California USA, www.graphpad.com). LN1 concentrations were deduced trough regression analysis.

#### Surface wettability

Static contact angle was measured on film samples at room temperature, using 5 μL DIW droplets by a video contact angle system in a KSV instrument equipped with a CAM 200 software for data acquisition. Sessile drop method was applied. For each sample, three measurements on different surface zones were averaged. Data are reported as mean value ± standard deviation.

#### *In vitro* degradation tests

Hydrolytic and enzymatic degradation tests using lipase (from porcine pancreas, Sigma-Aldrich, Italy) were performed weekly on PU and PU-LN1 scaffolds (6 mm diameter) up to 8 weeks. Degradation tests were carried out at 37°C under stirring at 50 rpm. The samples were placed in vials containing 0.1 ml PBS (pH 7.4) per mg sample in the case of hydrolytic tests, and 0.3 mg/mL lipase in PBS in the case of enzymatic tests. The degradation medium was renewed every 3 days. At each time point, three samples were withdrawn, washed with DIW and then dried at 37°C. Weight loss percentage was measured according to the formula:

$${\%}_{weight\;loss}=\frac{W_{0}-W}{W_{0}}∙100$$ (1)

where W_0_ is sample initial weight and W is the weight at each time point during the test.

At each time point, M_n_ loss percentage was also measured:

$${\%}_{M_{n}\;loss}=\frac{M_{n0}-M_{n}}{M_{n0}}∙100$$ (2)

where M_n0_ is the initial number average molecular weight, while M_n_ is the number average molecular weight at each time during the test. M_n_ was evaluated by Size Exclusion Chromatography (SEC; Agilent Technologies 1200 Series, USA), according to a previously published protocol [30]. Every two weeks, sample surface and cross section were also analyzed by SEM (LEO 1450VP). All samples were gold-coated before analysis and micrographs were taken with a beam voltage of 20 kV and a magnification of 80x and 300x for surfaces and 350x for cross sections.

#### Cytotoxicity of degradation products

Pristine PU scaffolds (6 mm diameter) were placed in vials containing 0.1 mL lipase solution (0.3 mg/mL in PBS) per mg and incubated at 37°C. Every three days, the degradation medium was changed, collected, and stored at -20°C. After complete degradation, the collected medium was thawed and lipase was deactivated by treating degradation medium for 2 hours at 65°C. PU degradation products were dehydrated by freeze drying (Martin Christ ALPHA 2–4 LSC) for 48 h. Powders were solubilized in cell medium and filtered in 0.22 μm membrane filters (Millex 33 mm Sterile Filter Unit with Millipore Express PES Membrane). 2×10^3^ NIH-3T3 mouse embryonic fibroblasts were seeded on 96-well plates in DMEM (Thermofisher, Italy), supplemented with 10% fetal bovine serum (FBS; MANUF), 100 U/mL penicillin (MANUF), and 100 μg/mL streptomycin (Sigma Aldrich, Italy) with or without 0.1 mg/mL PU degradation products. At each time point (0, 2, 4, 6 days), cells were washed with PBS to remove the medium, then 300 μL of the Cell Titer Blue/cell culture medium mixture (20% v/v) was added to each sample. Cell Titer Blue assay was performed following manufacturer’s recommendations. After 30 min, 100 μL of supernatant from each well were transferred to a new 96-well plate and fluorescence was measured using a GloMax-Multidetection System (Promega) at excitation wavelength (λ_ex_) of 525 nm and emission wavelength (λ_em_) of 580–640 nm.

### *In vitro* cell tests

#### Isolation of cardiac progenitor cells

Cardiac tissue samples were obtained from the atria of pathological hearts, which were explanted from patients with end-stage heart failure due to ischemic cardiomyopathy (n = 22, mean age 55.8 ± 3.1 years, 16 males, 6 females, mean ejection fraction 25 ± 1%) as a part of heart transplantation procedures. Specimens were collected without patient identifiers in accordance with the requirements approved by Monaldi Hospital and in conformity with the principles outlined in the Declaration of Helsinki. The samples were collected and anonymously provided by Monaldi Hospital (Naples, Italy). Cardiac tissue samples were dissected, minced, and then enzymatically disaggregated by incubation in 0.25 trypsin and 0.1 (%w/v) collagenase II (Sigma Aldrich, Italy) for 30 min at 37°C. Enzyme activity was stopped by adding a double volume of Hank’s balanced salt solution (HBSS; Sigma-Aldrich) supplemented with 10% FBS (Sigma Aldrich, Italy). Next, the tissue suspension was further disaggregated by pipetting and tissue debris and cardiomyocytes were removed by sequential centrifugation at 100 ×*g* for 2 min, passage through a 20 μm sieve, and centrifugation at 400 ×*g* for 5 min. Cells were then seeded on culture plates in DMEM/Nutrient Mixture F-12 (DMEM/F-12, Sigma-Aldrich, Italy) supplemented with 10% FBS, basic fibroblast growth factor (Peprotech, Rocky Hill, NJ, USA), glutathione (Sigma-Aldrich, Italy), penicillin and streptomycin (Life Technologies, Paisley, UK). Cells were cultured for a period ranging from 1 to 2 weeks. Once cells reached 75% confluence, they were detached with 0.25% trypsin-ethylenediaminetetraacetic acid (trypsin-EDTA;, Sigma-Aldrich, Italy). Finally, CPCs were isolated from cell suspension by immuno-magnetic cell sorting with anti-human-CD117 microbeads (Miltenyi Biotec, Bergisch Gladbach, Germany).

#### Scaffold cell seeding

CPCs were seeded on PU, PU-LN1 and PU-G scaffolds, previously sterilized by UV exposure (20 min for each side), in 96-well plates at a density of 7×10^4^ cells/cm^2^. Constructs were observed under an inverted phase contrast microscope (CKX41; Olympus Italia, Segrate, Italy) equipped with a digital camera (Color View IIIu Soft Imaging System, Muenster, Germany) during *in vitro* cell tests (7, 14 and 21 days).

#### Scaffold processing for microscopy

CPCs cultured on the scaffolds for 7, 14 and 21 days were fixed in 4% paraformaldehyde (PFA; Merck, Germany) for 20 min at room temperature, incubated with 30% sucrose (Sigma-Aldrich) at 4°C overnight and embedded in Tissue Freezing Medium (TFM, Leica Biosystems, Italy) in disposable molds (Bio-Optica, Italy). For quick freezing, molds were immersed in liquid nitrogen until TFM turned solid white and then stored at -80°C until cryosectioning. Slices of 10 μm were cut in a cryostat at -20°C and cryosections were collected on a microscope slide.

#### CPC immunofluorescence staining

Sections were incubated with primary anti-human antibodies against Ki67 (mouse monoclonal, Leica Microsystems), followed by secondary antibodies conjugated with fluorescein or rhodamine. Actin was revealed with FITC-labelled phalloidin (Jackson ImmunoResearch Europe, UK) and nuclei were counterstained with 4',6-diamidino-2-phenylindole (DAPI; Merck Millipore, Germany) or propidium iodide (Sigma-Aldrich, Italy). DAPI staining was also used for the identification of apoptotic bodies in apoptosis evaluation.

#### Morphological analysis

CPC adhesion on scaffolds was analyzed by SEM at 7 and 14 days, following a reported procedure [43, 44]. Briefly, constructs were fixed in 3% glutaraldehyde solution (Sigma-Aldrich, Italy) for 15 min at room temperature, followed by post-fixation with 1% osmium tetroxide (Sigma-Aldrich, Italy) for 15 min. After washing in PBS, specimens were dehydrated by graded alcohol series, followed by critical point drying (Emitech K850, Quorum Technologies, UK). Specimens were mounted on aluminium stubs with adhesive carbon tape and gold-sputtered prior to observation performed with an acceleration voltage of 5 kV at a working distance of 13 mm.

#### Gene expression analysis by RT-PCR

Total RNA was extracted from CPCs cultured on scaffolds for 7, 14 and 21 days using RNeasy RNA Isolation kit (QIAGEN, Italy), following the manufacturer’s protocol. The isolated RNA was dissolved in RNase-free water and the final concentration of RNA was determined in a Qubit fluorometer (Invitrogen, USA). A total amount of 100 ng RNA was reverse-transcribed into cDNA with QuantiTect Reverse Trascription Kit (QIAGEN, Germany). All primers (Table 1) were designed with Primer3 software [45]. RT-PCR was performed using RealMasterMix SYBR ROX (5Prime, Germany) according to the manufacturer’s protocol. DNA amplification (10 ng of starting cDNA) was carried out using Mastercycler ep realplex 4S (Eppendorf, Italy). The thermal cycling conditions included an initial denaturation for 2 min at 95°C and 40 cycles consisting of a denaturation step at 95°C for 15 s, an annealing step at 58°C for 15 s and an extension step for 30 s at 68°C. The binding of the fluorescence dye SYBR Green I (5Prime) to double-stranded DNA was measured. Samples were tested in triplicate with the housekeeping genes (GAPDH and RPL13A) to correct for variations in RNA quality and quantity. Melt curve analyses were conducted to assess uniformity of product formation, primer dimers formation and amplification of non-specific products. Linearity and efficiency of PCR amplification were assessed by standard curves generated by increasing amounts of cDNA. Comparative quantification of target genes expression in the samples was performed based on cycle threshold (C_t_) normalized to housekeeping genes, using the ΔΔC_t_ method.

**Table 1:** Primers designed with Primer3 software.

Gene	Forward Primer	Reverse Primer	Amplicon Length (nt)
**GAPDH**	5’-CACCATCTTCCAGGAGCGAG-3’	5’-TCACGCCACAGTTTCCCGGA-3’	372
**NKX2.5**	5’-CCTCAACAGCTCCCTGAC-3’	5’-CTCATTGCACGCTGCATA-3’	162
**MEF2C**	5’-AGGCAGCAAGAATACGATGC-3’	5’-TACGGAAACCACTGGGGTAG-3’	88
**α-SA**	5’-TCGGGACCTCACTGACTA-3’	5’- GGGCTGGAAGAGTGTCTC-3’	282
**ETS1**	5’-TGGGGACATCTTATGGGAAC-3’	5’-TGGATAGGCTGGGTTGACTC-3’	88
**FVIII**	5’-CAGCCTCTACATCTCTCAGTT-3’	5’-ATGCGAAGAGTGCTGCGAATG-3’	210
**GATA6**	5’- GCCCCTCATCAAGCCGCAGAA-3’	5’- TCTCCCGCACCAGTCATCACC-3’	378
**SMA**	5’-CTGAGCGTGGCTATTCCTTC-3’	5’-TTCTCAAGGGAGGATGAGGA-3’	133

### *In vivo* tests

#### Animal model and implantation

Subcutaneous implantation of cell-free PU and PU-LN1 scaffolds was performed in 6–8-week-old female FVB mice obtained from Charles River Laboratories International, Inc.. *In vivo* tests were carried out with the aim to preliminarily investigate body reaction (e.g. inflammation, integration, vascularisation) to the implantation of the newly designed PU and PU-LN1 scaffolds. In order to perform the same tests on human CPC cellularized scaffolds, nude or immunosuppressed mice would have been required, thus allowing *in vivo* assessment of the designed matrices in an environment that poorly recapitulates the real *in vivo* situation, being immune response absent or suppressed.

Mice received humane care in compliance with the Italian law (DL-116, Jan. 27, 1992) and the Guide for the Care and Use of Laboratory Animals published by the US National Institutes of Health (NIH Publication No. 85–23, revised 1996). The study have been approved by the Bioethics Committee of the University of Torino in 2011. All efforts were made to minimize suffering. Intramuscular injections of 100 mg/kg ketamine (Pfizer, Italy) and 5 mg/kg xylazine (Bayer, Italy) were applied to anesthetize the animals. The dorsal area of the animals was shaved and sterilized with 70% ethanol solution. One incision was made on the back and a subcutaneous pocket was created on each side. Mice were randomly assigned to 4 different groups (PU 15 days, PU-LN1 15 days, PU 30 days, PU-LN1 30 days), and 3 mice per group were subjected to implantation. The scaffold was subcutaneously implanted into each pocket and incisions were closed with polypropylene sutures. Upon surgery, mice were monitored once a day, 6 days per week. Mice were kept under controlled light (12/12 light /dark cycle) at room temperature (22 ± 1°C; U. R: 55 ± 10%). Food and water were provided ad libitum. The analysis was performed in blind. At 15 and 30 days post-surgery, mice were euthanized with CO_2_ asphyxiation followed by cervical dislocation, and implants were harvested for analysis.

#### Histology and immunochemistry

The implants and the surrounding tissues were fixed in 4% PFA, dehydrated with graded ethanol series, embedded in paraffin, cut into 3-μm thick sections and stained with hematoxylin and eosin (H&E; Bio-Optica, Italy) [46]. Blood vessels in the tissues surrounding the implants were counted on H&E stained sections.

## Statistical analysis

Statistical analysis was performed using GraphPad Prism ver. 6.0 for Windows (GraphPad Software, La Jolla California USA, www.graphpad.com). One-way ANOVA analysis followed by Tukey’s multiple comparison test was used to compare results. Statistical significance of each comparison was assessed as reported by Boffito et al. [47]. Unpaired two-tailed *t* test was used when indicated.

## Results

### Morphological characterization of scaffolds

**Fig 1:**
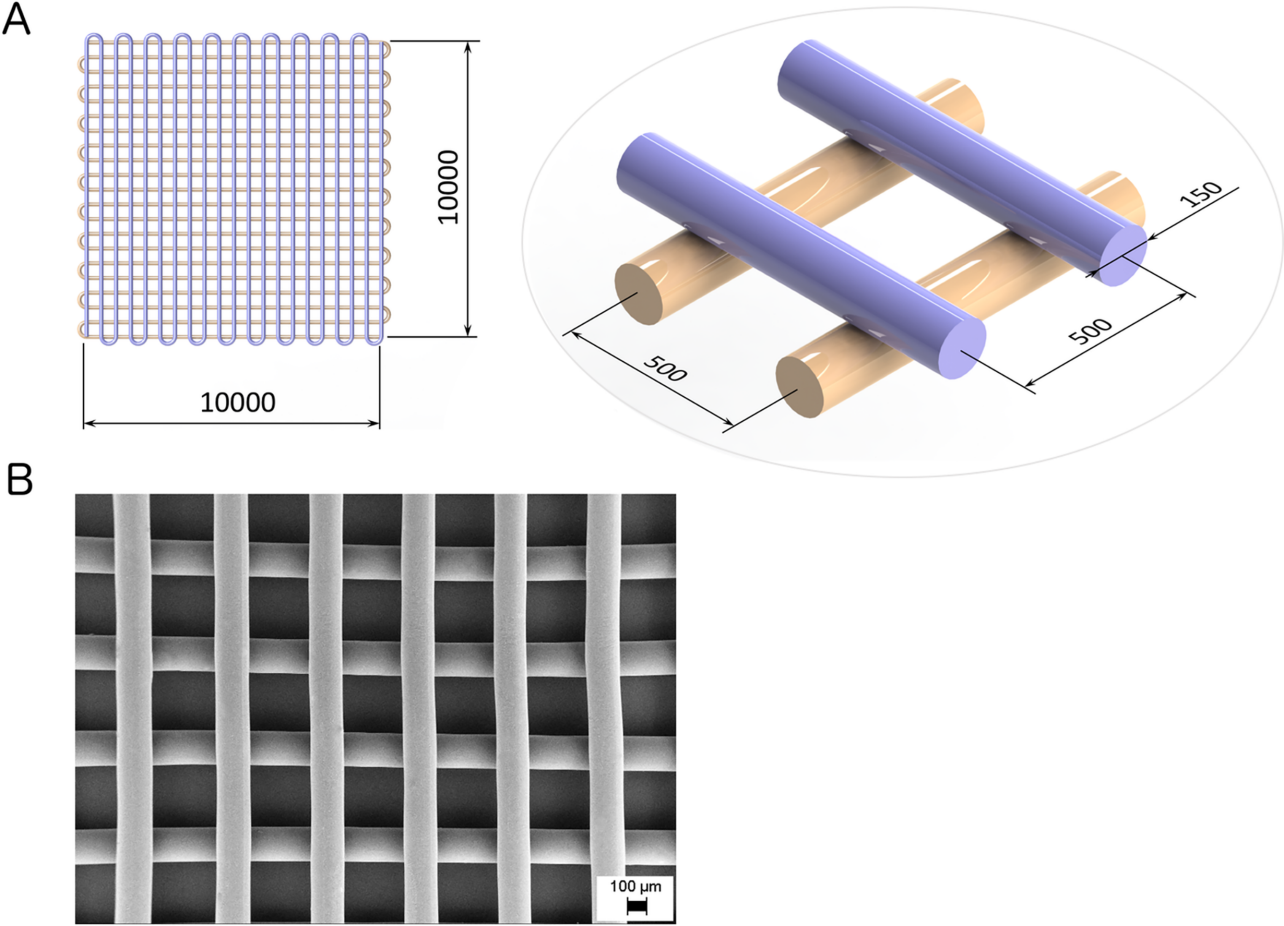
Scaffold design and micrograph. **(A)** Overall scaffold dimensions (top left) and a zoomed view of the two overlaying layers (bottom right) detailing fiber diameter (μm) and fiber-to-fiber distance (μm). **(B)** Representative FEG-SEM micrograph of an additively manufactured PU scaffold.

Fig 1A provides a schematic representation of the computer-generated scaffold geometry, while Fig 1B shows a representative Field Emission Gun Scanning Electron Microscopy (FEG-SEM) image of a PU scaffold. The suitability of PU to be melt-extruded in self-supporting multi-layered structures was previously demonstrated [35]. Results of image analysis on SEM micrographs showed a good agreement between the computer-generated geometry and the obtained scaffolds (mean fiber diameter: 155 ± 3 μm, mean spacing: 500 ± 4 μm). Such scaffold model geometry was here selected as it was characterized in our recent publication [35].

#### Physico-chemical characterization of surface functionalized PU films and scaffolds

**Fig 2:**
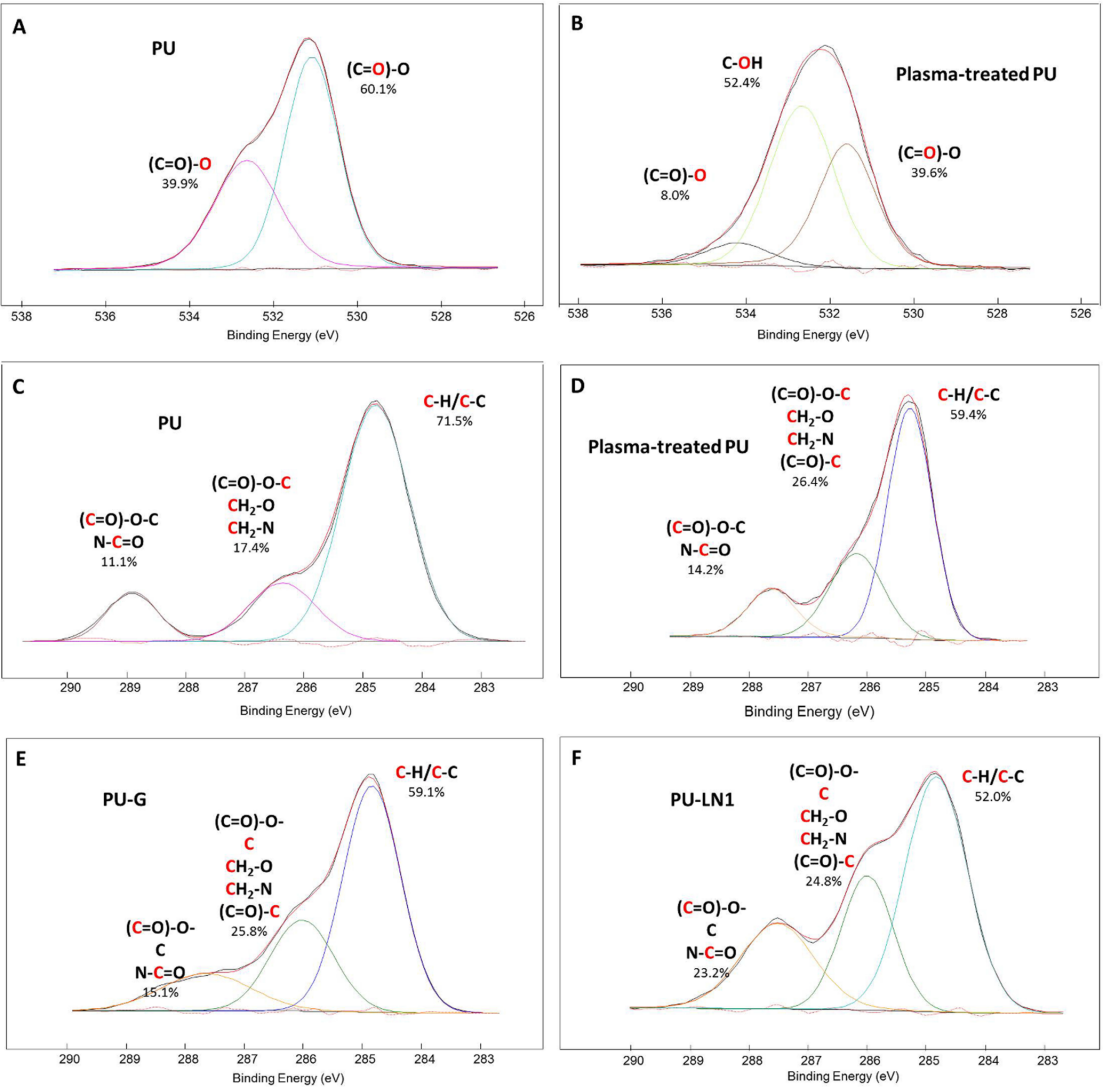
High resolution XPS spectra. **(A)** O1s of PU scaffolds; **(B)** O1s of plasma-treated PU scaffolds; **(C)** C1s of PU scaffolds; **(D)** C1s of plasma-treated scaffolds; **(E)** C1s of PU-G scaffolds and **(F)** C1s of PU-LN1 scaffolds.

Surface chemical properties of PU-based scaffolds were analyzed by X-ray Photoelectron Spectroscopy (XPS) analysis. Table 2 shows C, O and N elemental percentages and O/C and N/C ratios on PU-based samples. The elemental composition of PU scaffolds reflected their chemistry, characterized by the presence of ester groups in the poly(ε-caprolactone) (PCL) diol and urethane bonds along the PU chains. Plasma-treated PU showed a higher O percentage, attributed to the carboxylic groups of covalently grafted and polymerized acrylic acid. The O1s peak (Fig 2A and 2B) showed that, in PU scaffolds, oxygen was due to carbonyl oxygen O-C = O* and ester/urethane oxygen *O-C = O in nearly 60:40 ratio. After plasma treatment, the O1s spectrum contained a third component for hydroxyl oxygen (C-O*-H) (52.4%), with carbonyl oxygen O-C = O* and ester/urethane oxygen *O-C = O being reduced to 39.6% and 8.0%, respectively, in agreement with previous papers [48, 49]. After protein grafting, N percentage increased especially in the case of LN1 grafting while O/C ratio only slightly decreased compared to plasma-treated PU scaffolds (Table 2).

**Table 2:** C, O and N percentages and O/C and N/C ratios, obtained from XPS analysis.

SAMPLE	Atomic percentage (%)				
	C1s	O1s	N1s	O/C	N/C
**PU**	71.0	27.6	1.4	0.39	0.02
**Plasma-treated PU**	67.0	27.5	2.4	0.41	0.04
**PU-LN1**	64.1	23.5	12.4	0.37	0.19
**PU-G**	70.2	24.3	5.5	0.35	0.08

The higher N percentage of PU-LN1 scaffolds *vs*. PU-G scaffolds can be attributed to the higher amount of nitrogen rich residues (Asp, Glu, His, and Lys) of LN1 *vs*. G [50]. The C1s core-level spectra of unmodified and modified PU (Fig 2C, 2D, 2E and 2F) were deconvoluted into three main peaks at: 284.8 eV for carbon or carbon-hydrogen bonds (-C-C, -C-H), 286.0–286.4 eV for carbon-nitrogen or carbon-oxygen bonds (C-N, C-O), and 287.8–288.9 eV for carbonyl-like species (-COO-, C = O, -COOH at 288.9 eV, (C = O)-NH at 287.8 eV) [40, 42, 48, 49, 51]. In the C1s peak of plasma-treated PU, the contribution of C-C and C-H bonds decreased and those of carbonyl and C-O bonds increased, suggesting successful acrylic acid grafting/polymerization on the PU surface (Fig 2C and 2D). After protein grafting, the contribution of carbonyl groups increased (especially in the case of PU-LN1 scaffolds) and slightly shifted to lower eV (from 288.4 eV for plasma-treated PU scaffolds to 288.1 eV and 287.8 eV for PU-G and PU-LN1 scaffolds, respectively) suggesting that the amount of N-C* = O groups increased due to protein grafting [40, 48, 49, 51].

**Fig 3:**
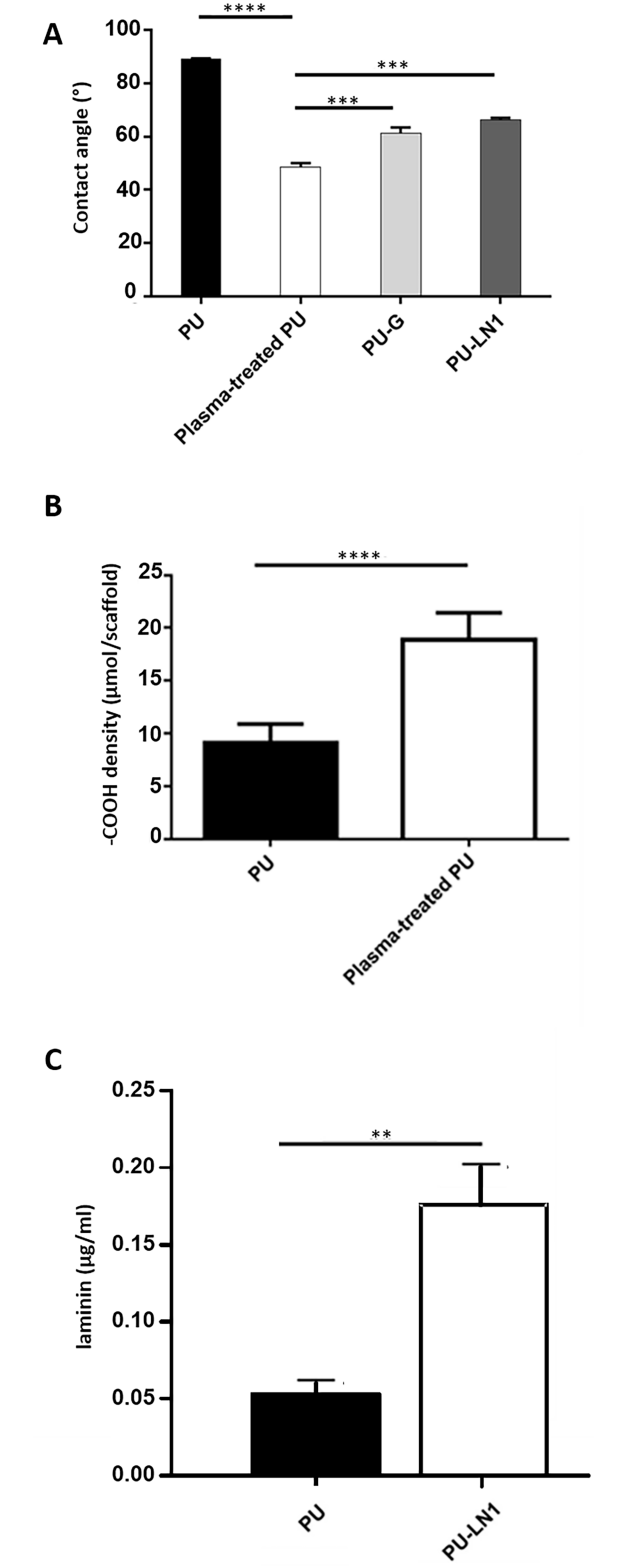
Characterization of functionalization steps. **(A)** Static contact angle values of PU (PU), plasma-treated PU (plasma-treated PU) and protein functionalized PU (PU-G and PU-LN1) films (*n* = 3; **** p<0.0001; *** p<0.001). **(B)** Colorimetric quantification of–COOH surface density in PU and plasma-treated PU scaffolds by TBO assay (*n* = 3, **** p<0.0001). **(C)** Quantification of LN1 grafting by ELISA assay: mean values of deduced LN1 concentrations measured on PU and PU-LN1 scaffolds (*n* = 3). Unpaired two-tailed *t* test was used for statistical analysis of data (**p < 0.01; ****p<0.0001).

Due to their different surface composition, PU-based samples also showed differences in surface wettability. Static contact angle analysis performed on PU-based films is reported in Fig 3A. PU films showed a contact angle of 89.1 ± 0.1°. After plasma treatment with acrylic acid, the static contact angle significantly decreased to 48.6 ± 1.5° confirming the grafting with hydrophilic acrylic acid functionalities. Grafting with G or LN1 significantly increased the contact angle to 61.3 ± 2.1° and 66.3 ± 0.8°, respectively. No significant differences in wettability were observed between PU-G and PU-LN1.

The successful introduction of–COOH groups by acrylic acid plasma treatment of PU scaffolds was further proved by Toluidine Blue O (TBO) colorimetric assay. After overnight incubation in TBO solution, plasma-treated PU samples showed an intense blue color (S1 Fig) associated with a significantly higher–COOH density (μmol/scaffold) *vs*. the control PU scaffolds, on which a lower density of TBO molecules was physically absorbed (Fig 3B). Scaffold functionalization with LN1 was analyzed by ELISA assay (Fig 3C). An ELISA standard curve (S2 Fig) was used to quantify LN1 amount on PU and PU-LN1 scaffolds through a regression analysis. Data indicated a significant difference in the presence of LN1 on PU-LN1 and PU scaffolds, demonstrating successful functionalization of PU-LN1 scaffolds.

### *In vitro* cell tests results

#### CPC adhesion on scaffolds

**Fig 4:**
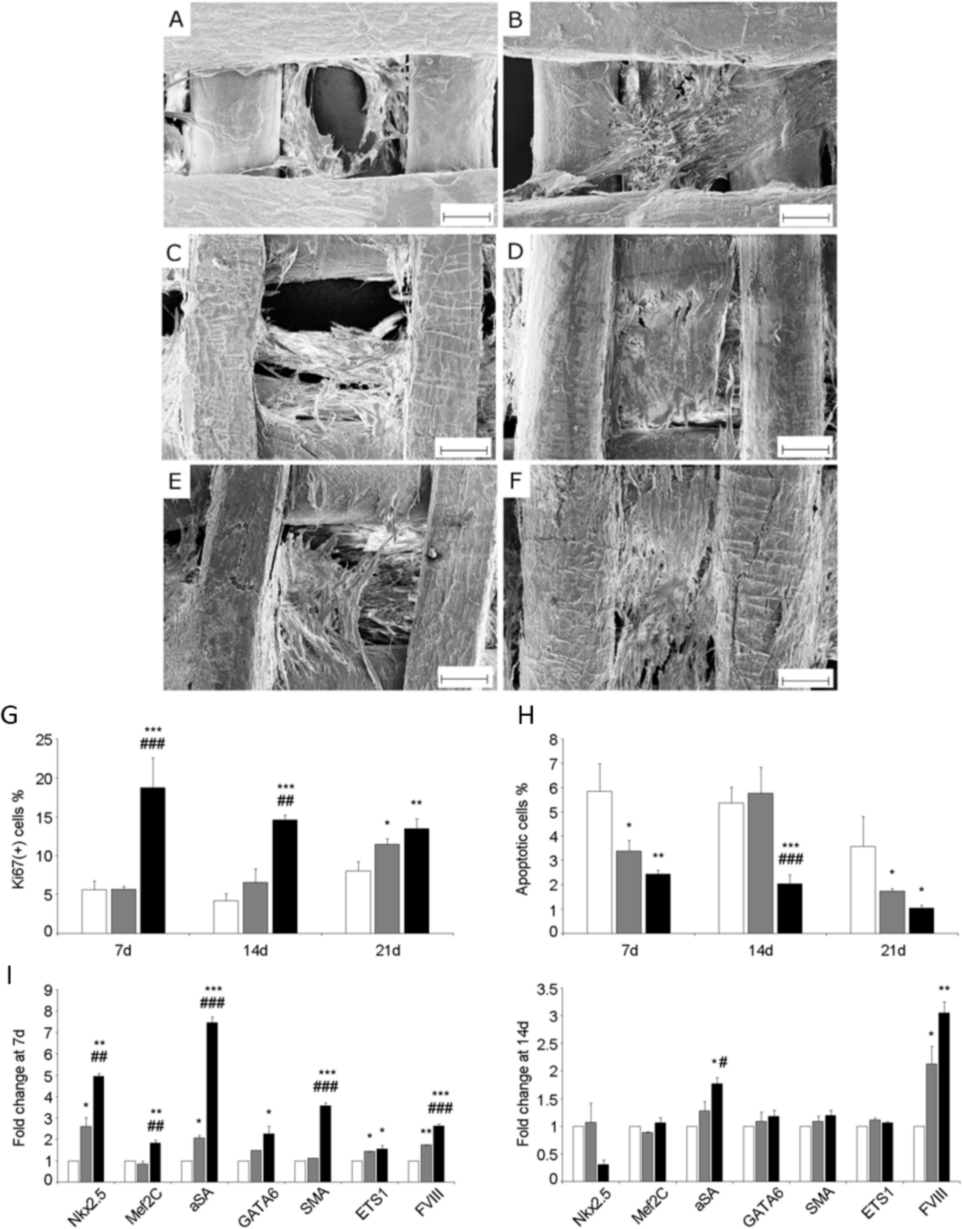
CPCs cultured on bare and surface-functionalized PU scaffolds: Cell morphology, proliferation, apoptosis and gene expression. SEM micrographs of PU-based scaffolds cultured with human CPCs for 7 (on the left) and 14 days (on the right): **(A, B)** PU, **(C, D)** PU-G, **(E, F)** PU-LN1. Scale bar: 100 μm. **(G)** Proliferation, **(H)** apoptosis and **(I)** gene expression of CPCs on PU scaffolds (control, white bars), PU-G scaffolds (grey bars) and PU-LN1 scaffolds (black bars) at different time points. *p<0.05, **p<0.01, ***p<0.001 *vs*. control, #p<0.05, ##p<0.01, ###p<0.001 *vs*. PU-G scaffolds.

Typically, 24 hours after seeding, cells adhered on the scaffolds and covered the filaments, spreading across the pores as observed by inverted phase contrast microscopy. Confocal image analysis revealed that the cells stretched out in three dimensions, among the fibers in the same and in the adjacent scaffold layers (S3 Fig). At each culture time, SEM micrographs (Fig 4A, 4B, 4C, 4D, 4E and 4F) showed higher cell engraftment on PU-G and PU-LN1 scaffolds *vs*. PU scaffolds. After 14 days, cells spread on the scaffold filaments, completely filling the pores. SEM analysis did not show significant differences between PU-LN1 and PU-G scaffolds in terms of cellularization.

#### Proliferation and apoptosis of CPCs on scaffolds

Proliferation of CPCs on PU, PU-G and PU-LN1 scaffolds was assessed by identifying and quantifying actively cycling Ki67-positive cells. The proliferation-associated protein Ki67, which is expressed by cells from late G1, through S and G2, to M phases, but not in resting cells in G0 cell cycle stage, was present in the cell nuclei. At 7 days, the proportion of cycling cells reached the highest value on the PU-LN1 scaffolds (18.56 ± 2.77%), exceeding 3.3 times the rate of Ki67-positive cells on PU (5.60 ± 1.15%) and PU-G (5.66 ± 0.40%) scaffolds (n = 3, p<0.001). The rate of cycling cells in PU-LN1 scaffolds decreased gradually over time; however, it remained higher than for the other scaffolds at each time point (Fig 4G). At 7 days, apoptosis of CPCs cultured on PU-LN1 scaffolds was 2.4-fold lower (2.44 ± 0.17%) respect to PU scaffolds (5.85 ± 1.15%, p<0.01) and 1.4-fold lower respect to PU-G scaffolds (3.39 ± 0.45%; p = ns). These differences were sustained, if not augmented, with time. Indeed, at 21 days the apoptosis rates were 3.58 ± 1.24% in PU scaffolds, 1.73 ± 0.12% in PU-G scaffolds, and 1.05 ± 0.11% in PU-LN1 scaffolds (Fig 4H).

#### Gene expression of CPCs on scaffolds

The population of CD117-positive cells from adult human heart consists of CPCs at different stages of their differentiation. The expression of the transcription factors Nkx2.5 and Mef2C identifies putative cardiomyocyte progenitors. Cardiomyocyte precursors differ from progenitors by the expression of cytoplasmic proteins, such as α-sarcomeric actin (αSA). Endothelial and smooth muscle cell progenitors can be identified by the expression of the transcription factor ETS1 and GATA6, respectively, while the precursors of the respective cell lineages also express factor VIII (FVIII) or smooth muscle actin (SMA) in the cytoplasm. The effect of scaffold functionalization on CPC differentiation was evaluated through the expression of typical markers of primitive cardiomyocyte, endothelial cell and smooth muscle cell lineages by Real-Time Quantitative Reverse Transcription-Polymerase Chain Reaction (RT-PCR) (Fig 4I). Notably, at 7 days, the expression of markers of cardiomyocyte (MEF2C, αSA), endothelial (ETS1, FVIII) and smooth muscle cell lineage (GATA6 and SMA) was significantly higher in CPCs cultured on PU-LN1 scaffolds compared to other experimental groups (Fig 4I). These differences were attenuated at 14 days, as the differentiation of the CPCs proceeded, although at lower pace, also in the control group and on the PU-G scaffolds. Nonetheless, even at 14 days, the expression of mRNA for cytoplasmic proteins αSA and FVIII peaked in the presence of LN-1 and was 3.05 and 2.13-fold higher than on the PU and PU-G scaffolds, respectively (Fig 4I). At 21 days, the lineage-specific characteristics of CPCs were preserved and the expression of markers did not change significantly (data not shown). PU-LN1 scaffolds were selected for further characterizations, such as in vitro degradation tests and in vivo trials.

### *In vitro* degradation tests on PU and PU-LN1 scaffolds

**Fig 5:**
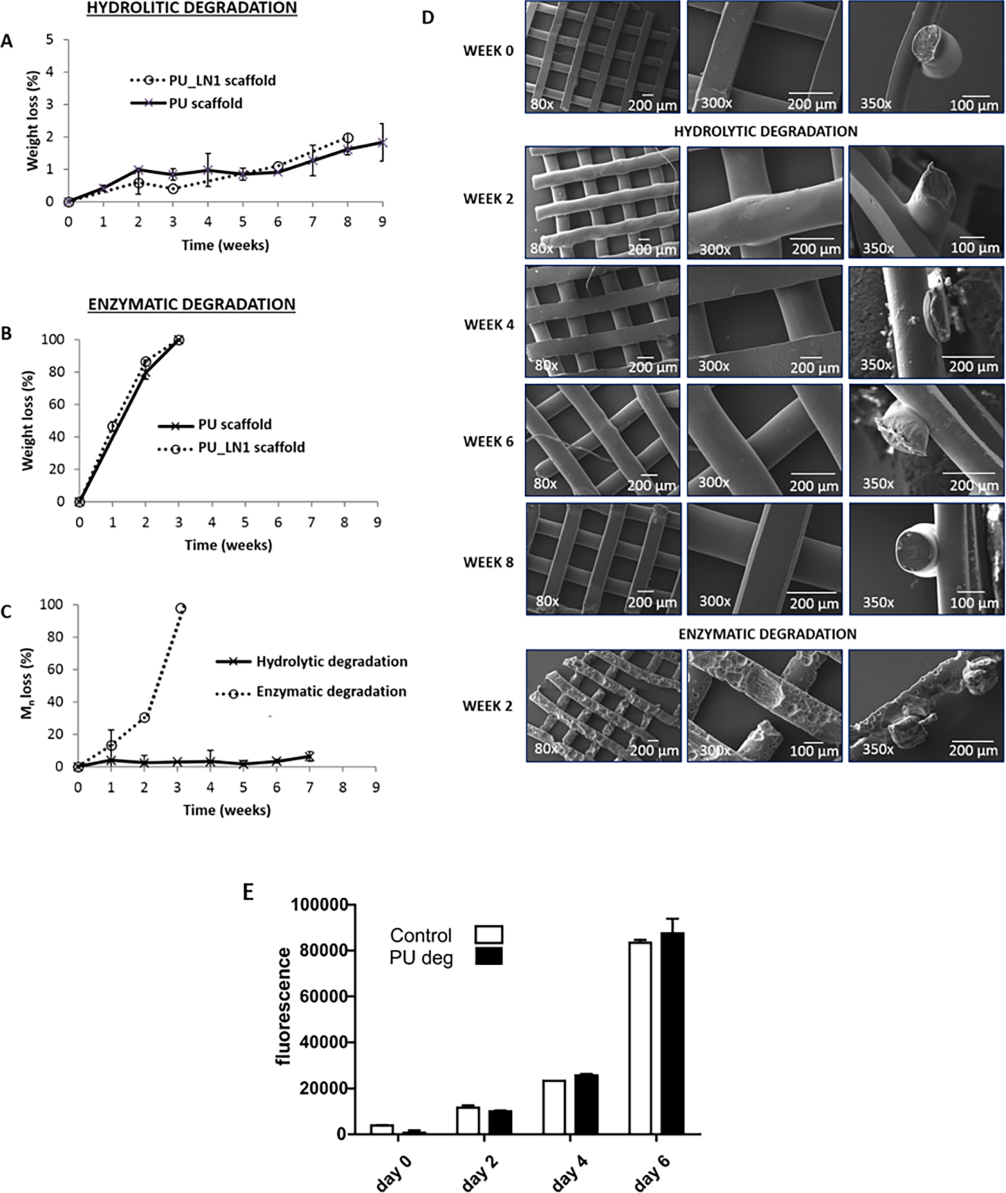
Hydrolytic and enzymatic degradation of bare and LN1-functionalized PU scaffolds: Weight loss, changes in morphology, loss of molecular weight and cytotoxicity of degradation products. Weight loss profiles of PU and PU-LN1 scaffolds undergoing **(A)** hydrolytic and **(B)** enzymatic degradation. **(C)** *M*_n_ loss profiles of PU scaffolds during hydrolytic and enzymatic degradation. **(D)** SEM micrographs of PU-LN1 scaffolds during hydrolytic and enzymatic degradation. **(E)** Viability of NIH-3T3 cells in the presence of Dulbecco’s Modified Eagle’s Medium (DMEM) containing PU degradation products (0.1 mg/mL) compared to control conditions, as evaluated by Cell Titer Blue assay (*n* = 4).

Hydrolytic and enzymatic degradation tests were performed on PU and PU-LN1 scaffolds to assess any effect of functionalization on degradation kinetics. Fig 5 shows the weight loss as a function of time for PU and PU-LN1 scaffolds in the case of hydrolytic (Fig 5A) and enzymatic degradation (Fig 5B), the Number Average Molecular Weight (*M*_n_) percentage loss as a function of time for PU scaffolds (Fig 5C), and SEM micrographs of the surface morphology of PU-LN1 scaffolds (Fig 5D) during both hydrolytic and enzymatic degradation. Enzymatic degradation was complete after 3 weeks (Fig 5B and 5C), while *M*_n_ loss due to hydrolytic degradation was only 5% after 8 weeks incubation in Phosphate Buffered Saline (PBS, pH 7.4) (Fig 5C). SEM analysis (Fig 5D) showed that PU-LN1 samples did not change their morphology during hydrolytic degradation tests, while samples incubated in the lipase-containing medium developed surface cracks. The viability of NIH-3T3 cells was evaluated in contact with the products of the complete degradation of PU scaffolds incubated in lipase medium following a previously developed method [52]. Results (Fig 5E) showed that no significant differences in cell viability were detected in the presence or in the absence of the degradation products.

### Results from *in vivo* tests

**Fig 6:**
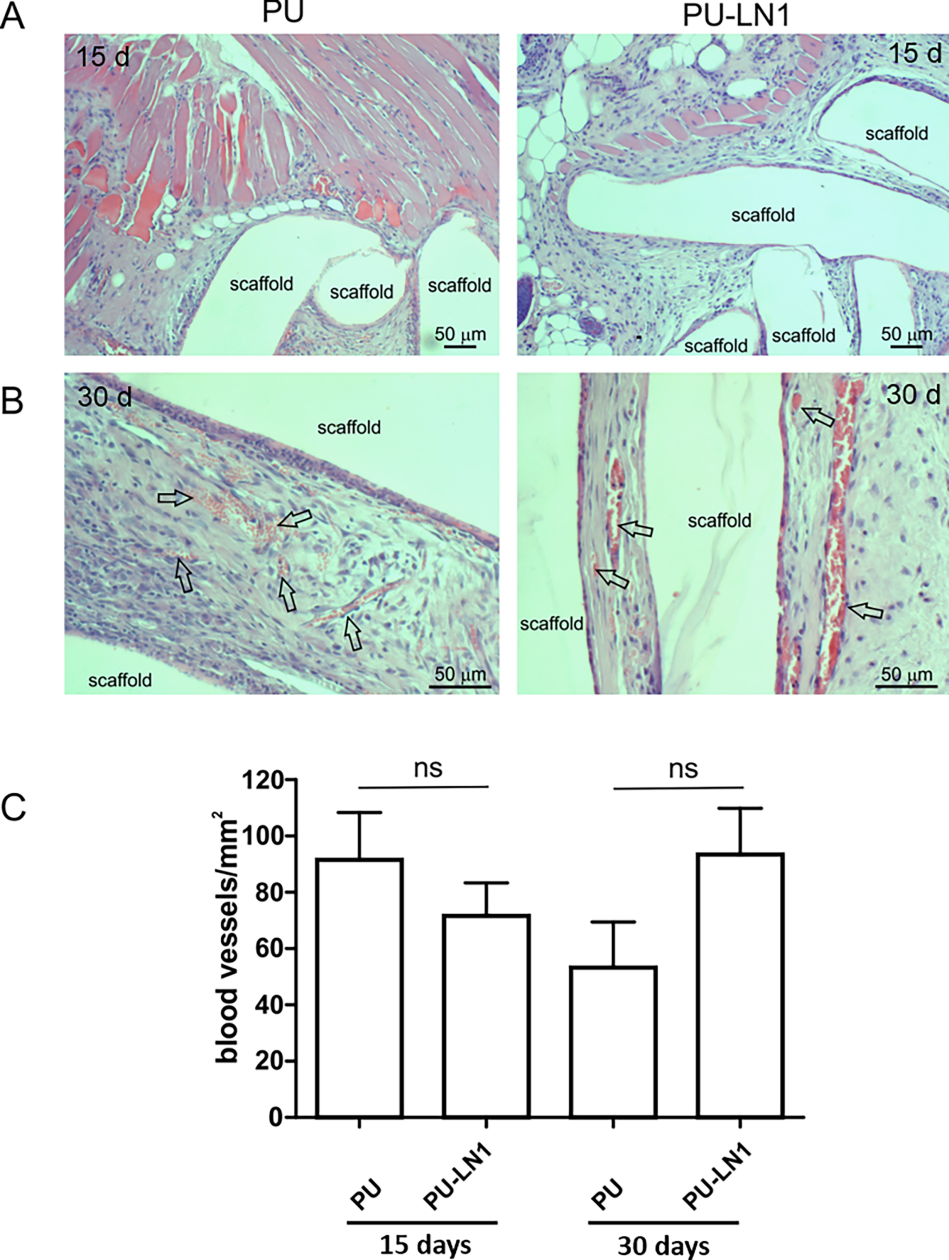
Results from *in vivo* tests carried out in mice: Histological analysis and quantification of blood vessels in the tissues surrounding the implants. Histological analysis by hematoxylin and eosin staining of PU and PU-LN1 scaffolds and surrounding tissues explanted 15 **(A)** and 30 **(B)** days following subcutaneous implantation in mice. Arrows indicate blood vessels (scale bar: 50 μm). **(C)** Number of blood vessels per mm^2^ in the tissues surrounding PU and PU-LN1 scaffolds, and explanted 15 and 30 days after subcutaneous implantation in mice. Unpaired two-tailed *t* test was used for statistical analysis.

PU and PU-LN1 scaffolds were subcutaneously implanted in mice to evaluate their biocompatibility and tissue response. All animals remained in good health during the experiments without signs of pain and macroscopic inflammation. After 15 and 30 days, mice were euthanized and the implants removed for further analysis. No signs of degradation or resorption were observed. Histological analysis demonstrated a good incorporation in the host tissues, with skeletal muscle, adipose and dermal tissues surrounding the scaffold fibers (Fig 6A). No differences were observed between PU and PU-LN1 scaffolds: no signs of inflammation, abscess formation, tissue necrosis or damage were present (Fig 6A). The number of blood vessels in the tissues surrounding the implants (Fig 6B) were not statistically different in both PU and PU-LN1 scaffolds (Fig 6C).

## Discussion

In this work, scaffolds mimicking the composition of the cardiac ECM were developed with the aim to positively affect CPC behavior, in terms of cell attachment, proliferation, viability and differentiation towards cardiomyocytes, endothelial and smooth muscle cells. In the literature, a number of scaffolds have been proposed for CPC-based therapy for myocardial regeneration, using synthetic materials [32–35], natural polymers [24], or their combinations [25]. The versatility of polyurethane chemistry for TE applications has been previously demonstrated [35, 36, 40, 47, 53]. A previously designed biocompatible PU [35] was here successfully processed by melt-extrusion AM, obtaining bi-layered scaffolds with a 0°/90° lay-down pattern (Fig 1). Selected fiber size and spacing allowed the deposition of self-supporting molten filaments during the fabrication of highly porous scaffolds. In our previous study, such scaffold geometry was found to support CPC adhesion and spreading [35]. However, CPCs could not proliferate on pristine PU scaffolds [35], suggesting the need for a biomimetic surface functionalization, that was investigated in the present work. A two-step plasma treatment was applied to PU scaffolds to surface graft/polymerize acrylic acid which was then exploited for carbodiimide-mediated conjugation of proteins [40]. Plasma treatment is a highly versatile tool for surface functionalization of polymeric substrates without affecting bulk material properties [54]. The literature also reports recently introduced challenging methods for polymer surface functionalization, such as mussel inspired protein-mediated surface functionalization [55–58], which offers the possibility to uniformly deposit biomolecules on complex surfaces, e.g. nanopatterned substrates, similarly to plasma approach. However, the detailed mechanism of DOPA-mediated surface functionalization is still under scientific debate, while plasma technology has already been introduced in industrial processes owing to its capability of treating objects with irregular and complicated shapes, the high control of all treatment parameters and steps, and its low cost.

Successful acrylic acid grafting/polymerization was demonstrated by XPS (Fig 2, Table 2) and static contact angle measurements (Fig 3A), and quantified by TBO assay (Fig 3B). Based on previous findings [27,28], LN1 was here selected as a target protein for scaffold surface functionalization, while G was chosen as a control molecule, as it is a low-cost protein, commonly used for cell culture experiments. Successful grafting of the proteins was confirmed by XPS (Fig 2, Table 2), static contact angle measurements, and ELISA assay (Fig 3A and 3C). PU-G and PU-LN1 scaffolds supported CPC adhesion (Fig 4, S3 Fig). Additionally, LN1 conjugated to the scaffold surface retained its ability to promote CPC proliferation (Fig 4G) and protect CPCs from apoptosis (Fig 4H), as previously demonstrated by Castaldo et al. for LN1 physically absorbed on Petri dishes [27]. PU-LN1 scaffolds were significantly superior to pristine PU and PU-G scaffolds in supporting CPC proliferation and protecting cells from apoptosis. Both PU-G and PU-LN1 scaffolds increased the differentiation commitment of CPCs to cardiomyocytes, smooth muscle and endothelial cells; however, CPC differentiation proceeded at a significantly higher pace when cultured on PU-LN1 scaffolds (Fig 4I). The effect of LN1 was particularly relevant considering that neither a differentiation culture medium nor a biomimetically-inspired scaffold architecture were additionally used to influence CPC behavior. Due to their superior properties, PU-LN1 scaffolds were selected and further analyzed for their *in vitro* degradation kinetics and *in vivo* tissue compatibility. Scaffold incubation in lipase solutions was here applied to demonstrate the complete biodegradability of the PU scaffolds and the cytocompatibility of their degradation products (Fig 5E) [59]. PU-LN1 and PU scaffolds underwent fast degradation by lipase through surface erosion (Fig 5D), while hydrolytic degradation was slow (Fig 5A, 5C and 5D) and proceeded through a bulk degradation mechanism (Fig 5D). Both mechanisms mainly involve the rupture of PCL ester bonds along the PU chains. Although LN1 functionalization increased the surface wettability compared to PU samples, its presence did not significantly affect the degradation rate. PU and PU-LN1 scaffolds subcutaneously implanted in mice were well incorporated in the host tissues and no signs of resorption or severe inflammation were observed up to 30 days (Fig 6A). Subcutaneous implantation of cardiac scaffolds has been previously performed as a preliminary test to analyze scaffold integration and vascularization [32, 60–62]. Particularly, respect to what found by Liu et al. for their non-cellularized nanofibrous poly(L-lactic) acid scaffolds [32], our non-cellularized PU-LN1 scaffolds supported long term angiogenesis (Fig 6B and 6C). Angiogenesis might be further improved by implanting cellularized PU-LN1 scaffolds due to a paracrine effect exerted by the cells, as previously demonstrated [32]. The obtained results open important perspectives in the field of CPC-based therapies, as they demonstrate enhanced *in vitro* survival, proliferation and differentiation of CPCs on PU scaffolds functionalized with a biomimetic protein of cardiac ECM (LN1) compared to scaffolds grafted with a non-cardiac mimetic cell-adhesive protein (G). To our knowledge, a chemical stimulation of human CPCs through the scaffold surface chemistry has not been exploited up to now and could be further combined with mechanical, electrical or topological cues with the aim to facilitate CPC differentiation into mature cardiomyocytes. Based on these results and previous literature [25], in the future, we will prepare PU-LN1 scaffolds more closely mimicking CPC niche microenvironment combining chemotactic signaling (LN1 functionalization) with biomimetic anisotropic architecture to analyze their influence on CPC maturation and differentiation, Importantly, such new scaffolds mimicking cardiac niche could be also exploited for *in vitro* studies of human CPCs behavior in a biomimetic 3D environment, which is more representative of the *in vivo* condition. Additionally, our results could have further impact by inspiring new approaches based on the use of LN1-functionalized scaffolds to facilitate *in vitro* differentiation of pluripotent stem cells into the cardiac phenotype or even the direct reprogramming of fibroblasts into cardiomyocytes.

## Conclusions

In this work, PU scaffolds were prepared by melt-extrusion AM technique and successfully surface functionalized with LN1 and G (as a control molecule for functionalization) by plasma-mediated grafting. Although both PU-G and PU-LN1 scaffolds showed increased cell density compared to pristine PU scaffolds, LN1 functionalization resulted in improved protection of CPCs from apoptosis and promotion of CPC proliferation, stimulating their differentiation to cardiomyocytes, endothelial cells and smooth muscle cells at a higher pace than G. PU and PU-LN1 scaffolds did not undergo significant hydrolytic degradation *in vitro* up to 8 weeks; however, their biodegradation ability was demonstrated by *in vitro* enzymatic degradation tests. Enzymatic degradation products were cytocompatible. Similarly, PU-LN1 and PU scaffolds, subcutaneously implanted in a mouse model, evoked a minimal inflammatory response and promoted angiogenesis. Hence, this work demonstrated the ability of PU-LN1 scaffolds to positively affect CPC behavior, being LN1 a key component of cardiac niche ECM. Results from this study were the first evidence of the possible application of PU-LN1 scaffolds as *in vitro* models of myocardial microenvironment with the aim to study CPC behavior, or as implantable patches for myocardial TE.

## Supporting information

S1 Fig Photos of scaffolds after TBO assay. Typical aspect of untreated PU control scaffolds (PU) and plasma treated PU scaffolds (Plasma-treated PU) after TBO colorimetric assay.
(TIF)

S2 Fig ELISA standard curve for LN1. A second order polynomial best-fit curve was used to generate a regression formula (R^2^ = 0.95). LN1 concentrations were deduced through regression using GraphPad program.
(TIF)

S3 Fig Morphology of CPC cultured on pristine and surface-functionalized PU scaffolds for 7 days. Representative images of CPCs cultured for 7 days on PU **(A, D)**, PU-G **(B)** and PU-LN1 **(C)** scaffolds observed by phase contrast microscopy (A, B, C) and confocal microscopy after actin cytoskeleton (green) and nuclei (red) staining on PU scaffolds (D). Scale bar is 200 μm.
(TIF)

S1 File NC3Rs ARRIVE guidelines checklist. (DOCX)
