# Supplementary figures and images for: Surface functionalization of polyurethane scaffolds mimicking the myocardial microenvironment to support cardiac primitive cells
Source: PLoS One. 2018 Jul 6;13(7):e0199896. doi: 10.1371/journal.pone.0199896 (PMC6034803; doi:10.1371/journal.pone.0199896)

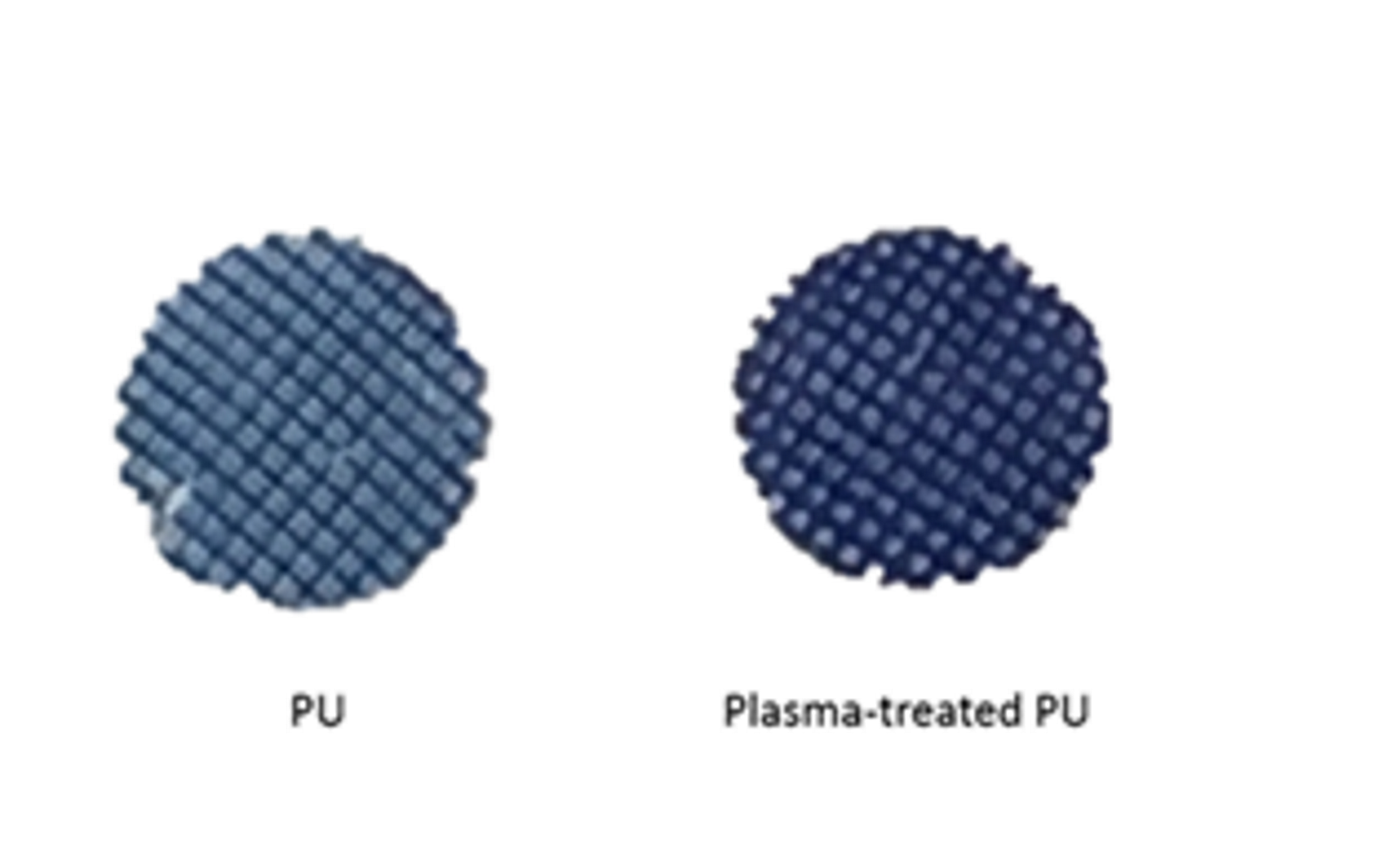

Supplement: S1 Fig — Typical aspect of untreated PU control scaffolds (PU) and plasma treated PU scaffolds (Plasma-treated PU) after TBO colorimetric assay. (TIF) [file pone.0199896.s001.tif]

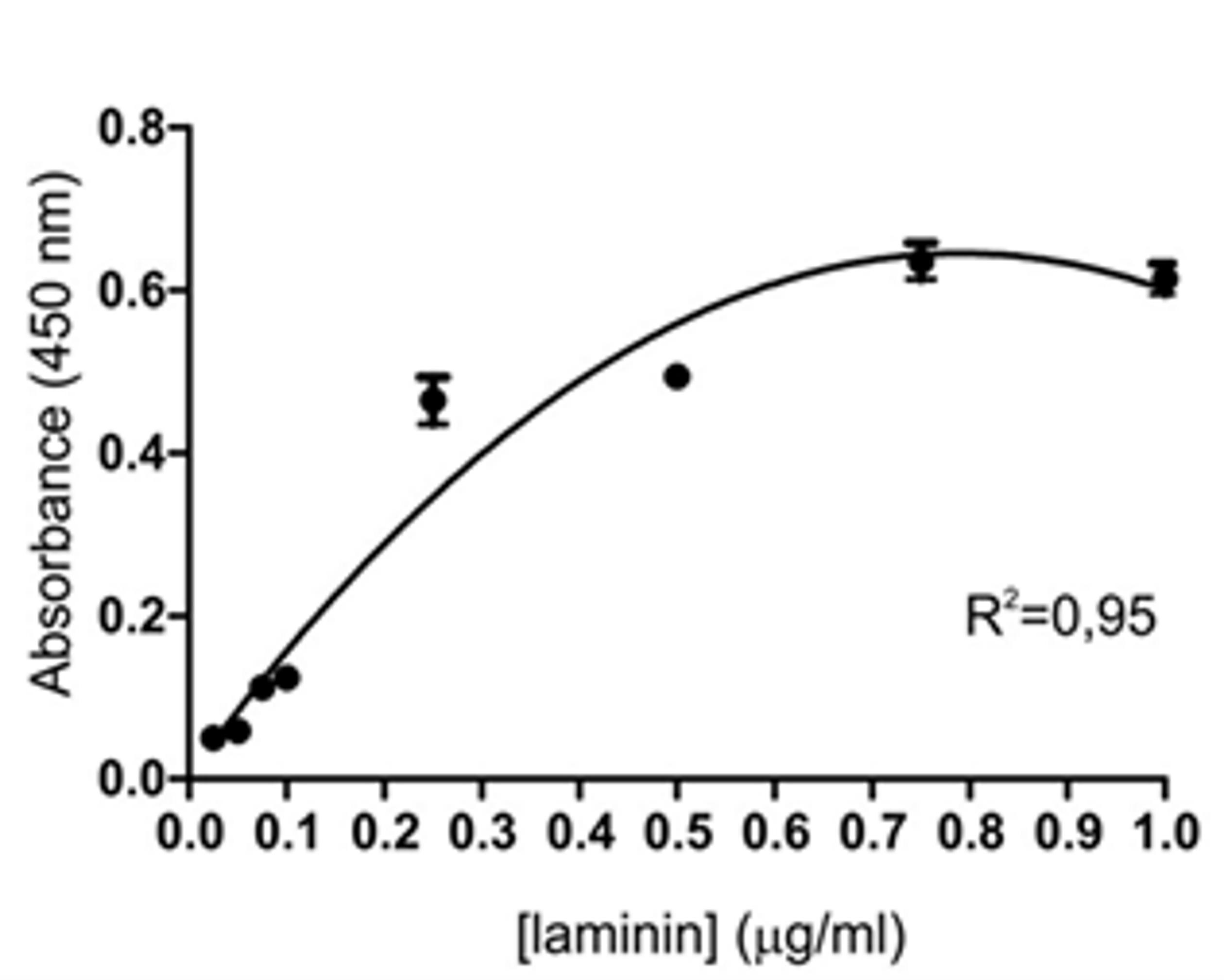

Supplement: S2 Fig — A second order polynomial best-fit curve was used to generate a regression formula (R2 = 0.95). LN1 concentrations were deduced through regression using GraphPad program. (TIF) [file pone.0199896.s002.tif]

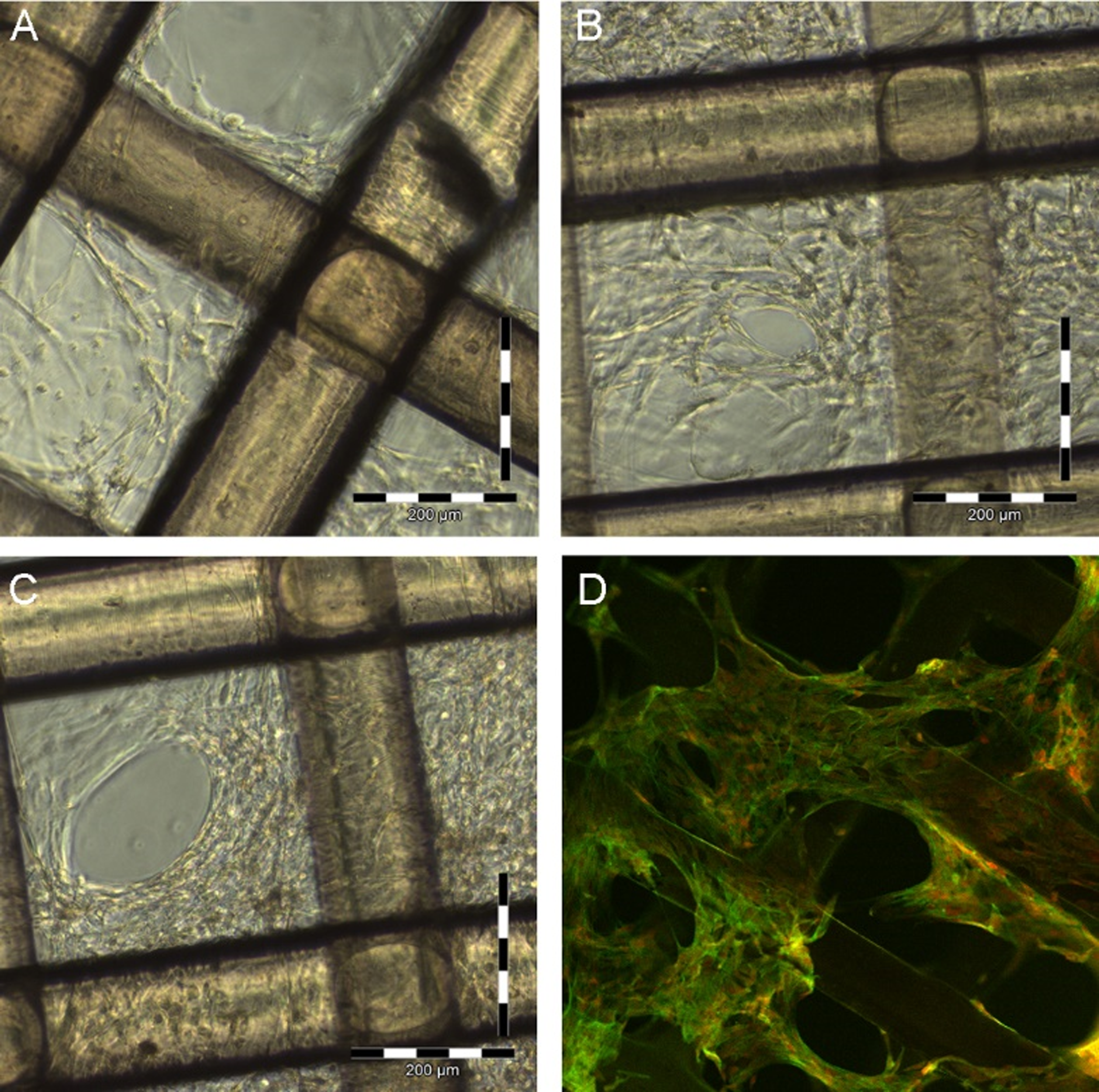

Supplement: S3 Fig — Representative images of CPCs cultured for 7 days on PU (A, D), PU-G (B) and PU-LN1 (C) scaffolds observed by phase contrast microscopy (A, B, C) and confocal microscopy after actin cytoskeleton (green) and nuclei (red) staining on PU scaffolds (D). Scale bar is 200 μm. (TIF) [file pone.0199896.s003.tif]
