# Supplementary material for: Surface functionalization of polyurethane scaffolds mimicking the myocardial microenvironment to support cardiac primitive cells
Source: PLoS One. 2018 Jul 6;13(7):e0199896. doi: 10.1371/journal.pone.0199896 (PMC6034803; doi:10.1371/journal.pone.0199896)

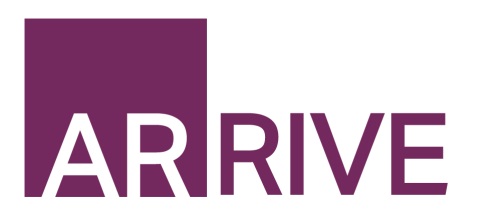


The ARRIVE Guidelines Checklist

Animal Research: Reporting In Vivo Experiments

Carol Kilkenny^1^, William J Browne^2^, Innes C Cuthill^3^, Michael Emerson^4^ and Douglas G Altman^5^

*^1^The National Centre for the Replacement, Refinement and Reduction of Animals in Research, London, UK, ^2^School of Veterinary Science, University of Bristol, Bristol, UK, ^3^School of Biological Sciences, University of Bristol, Bristol, UK, ^4^National Heart and Lung Institute, Imperial College London, UK, ^5^Centre for Statistics in Medicine, University of Oxford, Oxford, UK.*

|  | | ITEM | RECOMMENDATION | Section/ Paragraph |
| --- | --- | --- | --- | --- |
| 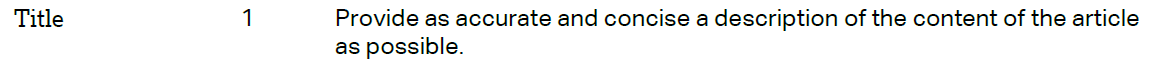 | | | Title |  |
| 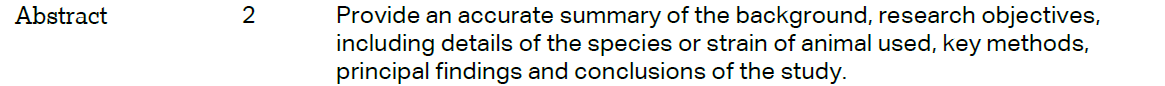 | | | Abstract |  |
| INTRODUCTION | | |  |  |
| 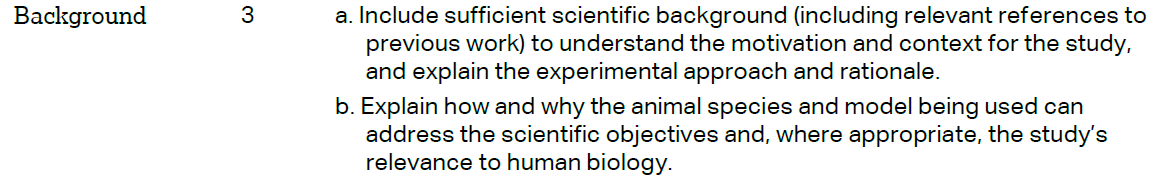 | | | Introduction  Introduction |  |
| 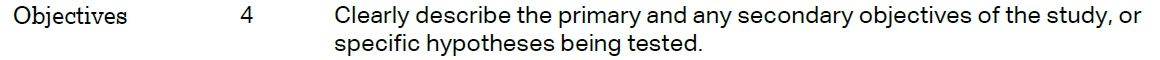 | | | Introduction |  |
| METHODS | | |  |  |
| 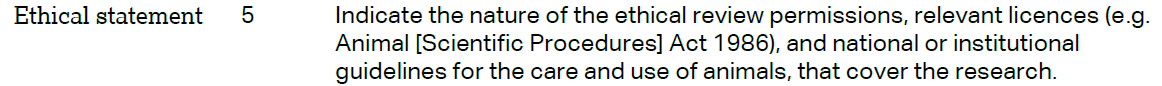 | | | “Animal model and implantation” |  |
| 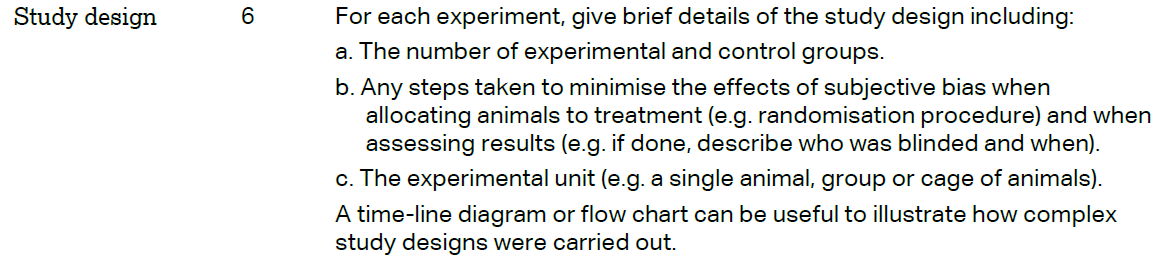 | | | “Animal model and implantation”  “Histology and immunochemistry” |  |
| 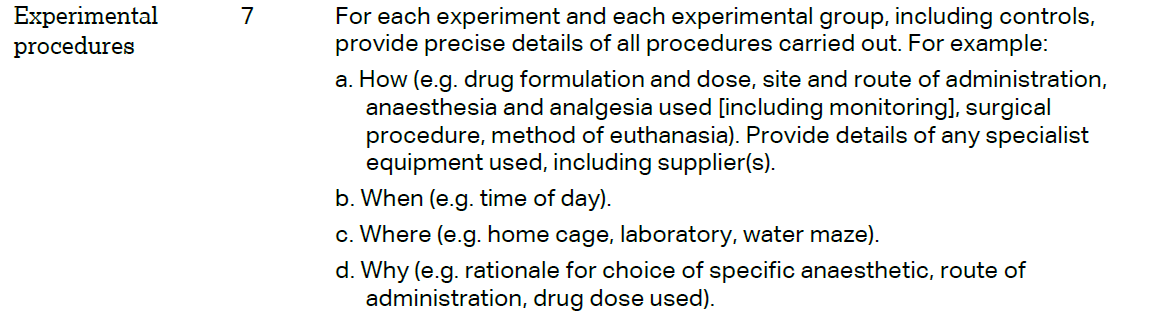 | | | “Animal model and implantation” |  |
| 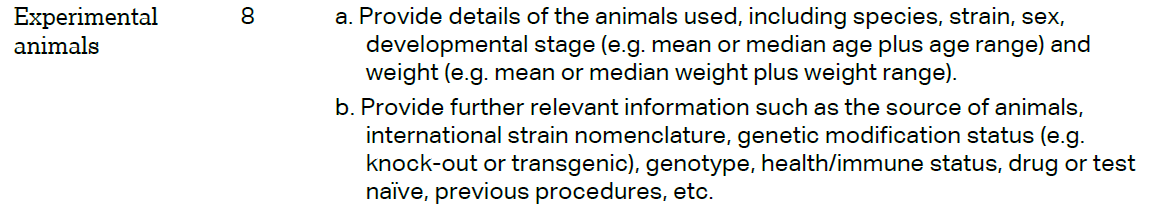 | | | “Animal model and implantation” |  |

The ARRIVE guidelines. Originally published in *PLoS Biology*, June 2010^1^

| 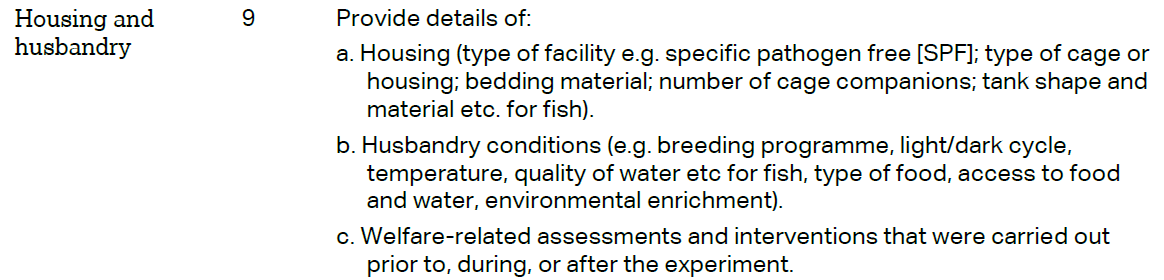 | “Animal model and implantation” | |
| --- | --- | --- |
| 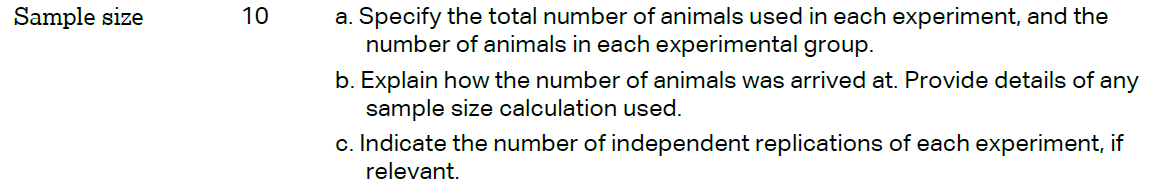 | “Animal model and implantation” | |
| 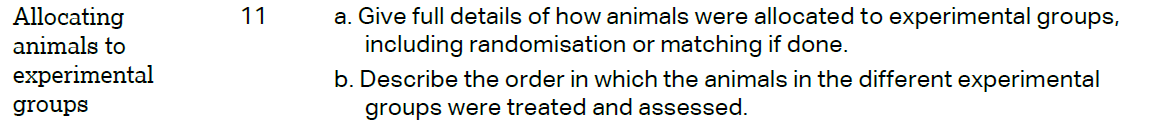 | “Animal model and implantation” | |
| 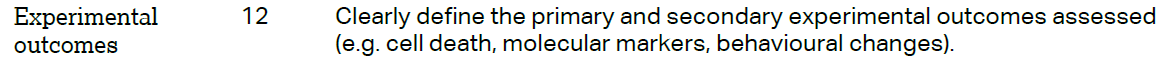 | “Results from in vivo tests” | |
| 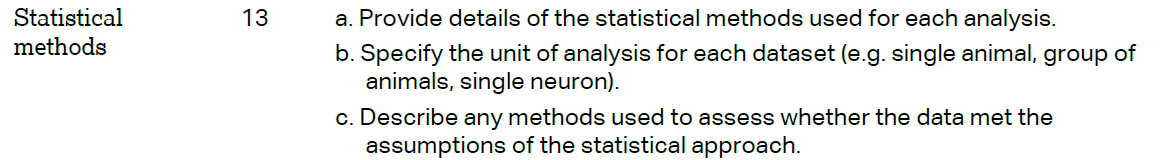 | “Statistical analysis” | |
| RESULTS |  | |
| 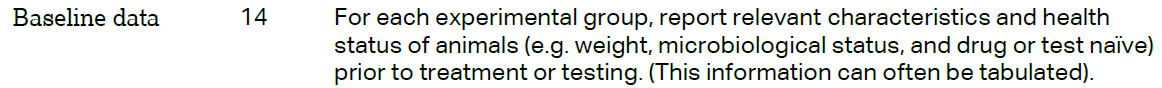 | “Animal model and implantation” | |
| 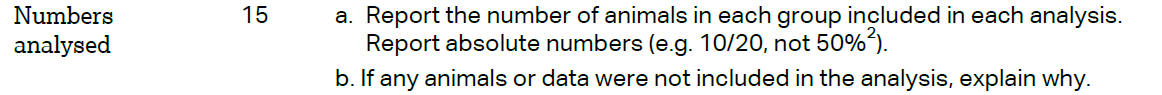 | “Animal model and implantation” | |
| 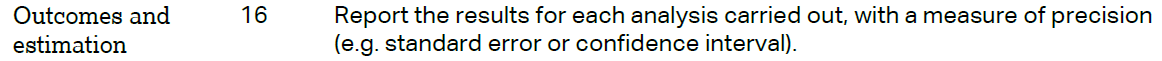 | “Results from in vivo tests” | |
| 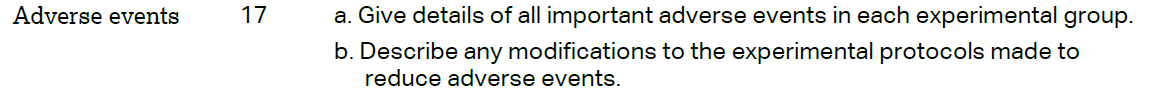 | “Animal model and implantation” | |
| DISCUSSION |  | |
| 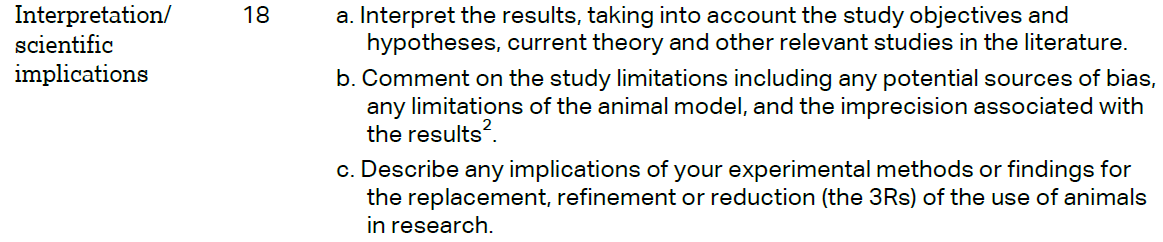 | “Discussion” | |
| 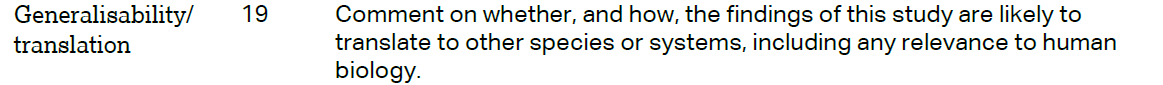 | “Discussion” | |
| 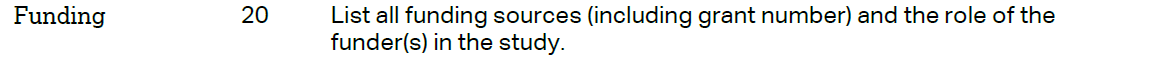 | | “Funding sources” |


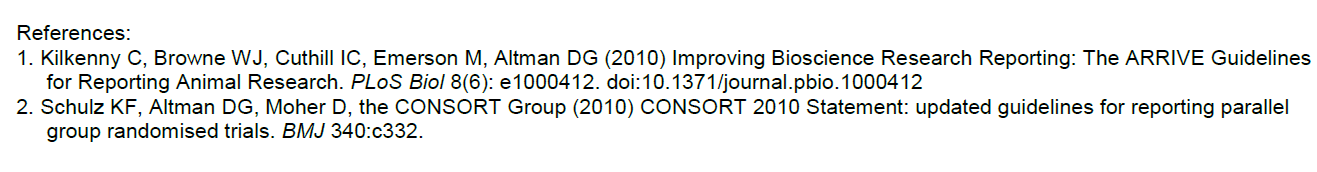

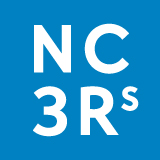

Supplement: S1 File — (DOCX) [file pone.0199896.s004.docx]
